# Supplementary material for: Predictors of shared decision-making among treatment-seeking emerging adults in primary care and community addiction and mental health settings: A cross-sectional study
Source: PLoS One. 2025 Nov 13;20(11):e0336598. doi: 10.1371/journal.pone.0336598 (PMC12614592; doi:10.1371/journal.pone.0336598)
Supplement: S1 File — (PDF) [file pone.0336598.s001.pdf]

Alberta Shared Decision Making Measurement Instrument (Health Care Provider)

This purpose of this tool is to gather information about shared decision making between you and your patient/client. A shared decision is one that you and patient/client make together, after considering options based on the best available evidence and your patient's /client's preferences. For the purposes of this tool, a health care provider is anyone who has a clinical appointment with a patient/client regarding his or her health, such as a physician, a nurse, a nurse practitioner, a dietician, a social worker, a mental health therapist, or an exercise specialist.

PAT ID:	
HCP ID:	

Instructions: Put an x in each row to indicate how strongly you agree or disagree.

	Strongly Agree	Mostly Agree	Moderately Agree	Slightly Agree	Mostly Disagree	Strongly Disagree	Not Applicable
Q1. My patient/client and I agreed on the main concern(s) and focus of the visit.							
Q2. My patient/client and I worked together to make a plan that addressed my patient's/client's preferences.							
Q3. The plan that my patient/client and I made considered his/her wishes and abilities.							
Q4. I checked that my patient/client understood his/her plan.							
Q5. I checked if my patient/client could follow the plan between now and our next appointment.							
Q6. My patient/client agreed with the plan we created.							

Please rate the shared decision making for this visit by placing an X next to the word that best describes your rating.

____ Excellent
____ Acceptable
____ Unacceptable
