# Supplementary material for: Predictors of shared decision-making among treatment-seeking emerging adults in primary care and community addiction and mental health settings: A cross-sectional study
Source: PLoS One. 2025 Nov 13;20(11):e0336598. doi: 10.1371/journal.pone.0336598 (PMC12614592; doi:10.1371/journal.pone.0336598)
Supplement: S2 File — (PDF) [file pone.0336598.s002.pdf]

ASK-MI6-PAT

	Strongly Agree	Mostly Agree	Moderately Agree	Slightly Agree	Mostly Disagree	Strongly Disagree	Not Applicable
Q1. My health care provider and I agreed on the main concern(s) and focus of the visit.							
Q2. My health care provider and I worked together to make a plan that addressed my preferences.							
Q3. The plan that my health care provider and I made considered my wishes and abilities.							
Q4. My health care provider checked that I understood the plan.							
Q5. My health care provider checked if I could follow the plan between now and my next appointment.							
Q6. I agreed with the plan my health care provider and I made.							

Please rate the shared decision-making for this visit by placing an X next to the word that best describes your rating.

____ Excellent
____ Acceptable
____ Unacceptable

Please indicate whether you involved family or friends in a discussion of your treatment options.

____ No
____ Yes
