# Supplementary material for: Predictors of shared decision-making among treatment-seeking emerging adults in primary care and community addiction and mental health settings: A cross-sectional study
Source: PLoS One. 2025 Nov 13;20(11):e0336598. doi: 10.1371/journal.pone.0336598 (PMC12614592; doi:10.1371/journal.pone.0336598)
Supplement: Attachment — Submitted filename: Marshall SDM Emerging Adult PLoS One RD 2024 Jul 23 Final.docx [file pone.0336598.s005.docx]

When submitting your revision, we need you to address these additional requirements.

1. Please ensure that your manuscript meets PLOS ONE's style requirements, including those for file naming. The PLOS ONE style templates can be found at

https://journals.plos.org/plosone/s/file?id=wjVg/PLOSOne_formatting_sample_main_body.pdf and <https://journals.plos.org/plosone/s/file?id=ba62/PLOSOne_formatting_sample_title_authors_affiliations.pdf>

We have double-checked the requirements and have used the style template as requested.

2. We note that the grant information you provided in the ‘Funding Information’ and ‘Financial

Disclosure’ sections do not match. When you resubmit, please ensure that you provide the correct grant numbers for the awards you received for your study in the ‘Funding Information’ section.

Thank you, this has been corrected in the system to state the following:

*“This research has been funded by the generous support of the Stollery Children’s Hospital Foundation through the Women and Children’s Health Research Institute. This research was also funded by the Faculty of Graduate Studies and Research (RES0033014) at the University of Alberta.”*

3. In your Data Availability statement, you have not specified where the minimal data set underlying the results described in your manuscript can be found. PLOS defines a study's minimal data set as the underlying data used to reach the conclusions drawn in the manuscript and any additional data required to replicate the reported study findings in their entirety. All PLOS journals require that the minimal data set be made fully available. For more information about our data policy, please see http://journals.plos.org/plosone/s/data-availability.

Upon re-submitting your revised manuscript, please upload your study’s minimal underlying data set as either Supporting Information files or to a stable, public repository and include the relevant URLs, DOIs, or accession numbers within your revised cover letter. For a list of acceptable repositories, please see

http://journals.plos.org/plosone/s/data-availability#loc-recommended-repositories. Any potentially identifying patient information must be fully anonymized.

Important: If there are ethical or legal restrictions to sharing your data publicly, please explain these restrictions in detail. Please see our guidelines for more information on what we consider unacceptable restrictions to publicly sharing data: http://journals.plos.org/plosone/s/data-availability#loc-

unacceptable-data-access-restrictions. Note that it is not acceptable for the authors to be the sole named individuals responsible for ensuring data access. We will update your Data Availability statement to reflect the information you provide in your cover letter.

We have confirmed with our ethics board that we are able to provide a minimal, deidentified dataset of the primary outcome measures which can be found in the supporting information.

4. We notice that your supplementary tables are included in the manuscript file. Please remove them and upload them with the file type 'Supporting Information'. Please ensure that each Supporting Information file has a legend listed in the manuscript after the references list.

We have removed the supplementary tables from the manuscript and have included them as supplementary information as instructed.

Additional Editor Comments:

Dear Drs. Marshall and Vohra,

Thank you for your submission to PLOS ONE. Your manuscript was sent to two expert reviewers, both provided thorough feedback and asked for major revisions. Their detailed feedback is attached.

Given my familiarity with the field of SDM in mental health, I would like to offer additional feedback specifically related to your Introduction and Discussion sections. Please ensure your arguments align with current and updated work in SDM in mental health. For example, when you write that SDM is associated with better satisfaction and adherence, you referenced SDM studies not in the mental health field, although there are several reviews about SDM intervention in mental health. Another example is in the Discussion section; the opening paragraph (and subsequent discussion) appears to inaccurately present SDM in mental health in the context of medication decisions only, neglecting the broader spectrum of SDM in mental health interventions, such as those in recovery, psychiatric rehabilitation, self determination, and social prescribing (e.g., employment, housing).

Thank you, we agree and have performed a major revision to the manuscript (almost entirely rewritten in many places) to be more up-to-date with current literature, inclusive of the broader spectrum of SDM in a variety of mental health interventions.

We have cited and discussed the following additional citations in the introduction and discussion.

Schauer, C., Everett, A., Del Vecchio, P., & Anderson, L. (2007). Promoting the value and practice of shared decision-making in mental health care. *Psychiatric Rehabilitation Journal*, *31*(1), 54.

Drake, R. E., Cimpean, D., & Torrey, W. C. (2009). Shared decision making in mental health: prospects for personalized medicine. *Dialogues in clinical neuroscience*, *11*(4), 455-463.

**Reviewers' comments:**

Reviewer's Responses to Questions

Comments to the Author

1. Is the manuscript technically sound, and do the data support the conclusions?

The manuscript must describe a technically sound piece of scientific research with data that supports the conclusions. Experiments must have been conducted rigorously, with appropriate controls, replication, and sample sizes. The conclusions must be drawn appropriately based on the data presented.

Reviewer #1: Partly

Reviewer #2: Partly

2. Has the statistical analysis been performed appropriately and rigorously?

Reviewer #1: No

Reviewer #2: Yes

3. Have the authors made all data underlying the findings in their manuscript fully available?

The PLOS Data policy requires authors to make all data underlying the findings described in their manuscript fully available without restriction, with rare exception (please refer to the Data Availability Statement in the manuscript PDF file). The data should be provided as part of the manuscript or its supporting information, or deposited to a public repository. For example, in addition to summary statistics, the data points behind means, medians and variance measures should be available. If there are restrictions on publicly sharing data—e.g. participant privacy or use of data from a third party—those must be specified.

Reviewer #1: Yes

Reviewer #2: No

4. Is the manuscript presented in an intelligible fashion and written in standard English?

PLOS ONE does not copyedit accepted manuscripts, so the language in submitted articles must be clear, correct, and unambiguous. Any typographical or grammatical errors should be corrected at revision, so please note any specific errors here.

Reviewer #1: Yes

Reviewer #2: Yes

5. Review Comments to the Author

Please use the space provided to explain your answers to the questions above. You may also include additional comments for the author, including concerns about dual publication, research ethics, or publication ethics. (Please upload your review as an attachment if it exceeds 20,000 characters)

**Reviewer #1:**

This manuscript explores the relationship between shared decision-making, patient health engagement, health-related quality of life, and anxiety and depression symptoms in emerging young adults. This topic is very important and understudied. However, the manuscript is limited by some analytical decisions and a lack of clarity around the research question and hypothesis. It is my recommendation that the paper could be substantially improved by re-analyzing the data as continuous rather than categorical and re-writing the introductory framing and conclusion of the paper.

My specific suggestions are below:

1. The authors provide a strong and clear discussion of the four measures used in this paper: ASK-MI, PHE-S, HADS, and HRQL. However, each of these measures is a continuous variable that they chose to dichotomize/make categorical. The authors do not describe the rationale for dichotomizing the data, and dichotomizing the data limits the potential variance that could be measured if the measures were included as continuous variables. The authors highlight in their limitations that there is limited variance, particularly in the ASK-MI, as none of the scores fell into the “unacceptable” range. Additionally, the decision to dichotomize the HRQL data based on the average national data could be questioned. The limited variance and dichotomized variables makes the results challenging to interpret. The paper would be substantially strengthened by using each of these variables in their initial continuous form.

We had dichotomized the data based on clinical significance to try and promote the clinical utility of the results (i.e., so the results may be more clinically meaningful). However, we agree that analyzing these variables on a continuous scale may provide some additional statistical power, and we have reanalyzed the data as suggested. To calculate dyadic SDM (ASK-MI) scores, we used the mean score between the healthcare provider and the patient.

2. The research questions and hypothesis proposed in the current study are not clear as a reader. In the “objectives” section of the abstract they note that they are exploring a) SDM association with PHE and HRQL and b) PHE associated with HRQL. They do not mention anxiety/depression in the objective or in the title of the paper. However, in the results section, they describe a main result of symptoms of anxiety/depression negatively related to PHE that was not part of a priori objective. Similarly, no hypothesis were provided as to the direction of the effect, and it is not clear what theory the authors have about these relationships a priori. Ideally, all null results will also be presented in the abstract.

We agree and have clarified our research questions with an accompanying hypothetical model, including a directional effect. Please note that this change, along with changing how SDM is measured, has resulted in a major revision of the manuscript with new results.

Revised research objective:

*“The primary objective of this study was to explore whether symptoms of anxiety, depression or HRQL were associated with the level of dyadic SDM reported after a clinical encounter among emerging adults who obtained treatment in a primary care or mental health setting in Alberta, Canada. We hypothesized that symptoms of anxiety or depression would be negatively associated with SDM (i.e., increased symptoms would yield decreased SDM ratings), and HRQL would be positively associated with SDM (i.e., increased HRQL would yield increased SDM ratings). Figure 1 displays the hypothetical model.* *In a post-hoc analysis, we explored whether patient age is associated with dyadic SDM. Here, we hypothesized age would be negatively associated with SDM.”*

3. Additionally, it may improve the clarity of the paper to include a theoretical model of how the four concepts (SDM, PHE, HRQL, A/D) relate to one another. It struck me that the initial objective proposed may describe a mediator model, and that the paper may also be strengthened by considering whether the authors are hoping to conduct a mediator model (SDM--> PHE--> HRQL). However, this does not include anxiety/depression, which appears to be a major interest for the authors.

Thank you for this comment. In response to our research question clarification, we have included a hypothetical model. Since we collected cross-sectional data, we have evidence of anxiety/depression during and shortly prior to the clinic visit and SDM scores corresponding to the clinic visit. We are unable to explore the impact of SDM on anxiety, depression, or HRQL after the appointment, but rather the reverse: exploring whether anxiety/depression and HRQL impact the level of SDM engagement that occurs during the clinic visit.

4. It would similarly be helpful to clarify what theoretical gap this paper is filling and how the sample relates to that. The authors write: “To our knowledge, no investigations have explored whether an association exists between SDM, PHE, and HRQL among emerging adults with anxiety and depression” (page 4), but my understanding is that their participants include a general sample of individuals receiving physical/mental health services with and without anxiety/depression.

Thank you, as a result of the above clarifications of the research question and analysis, we were able to fill a theoretical gap: (i) who SDM may be most appropriate for and (ii) what symptoms are barriers and facilitators for achieving SDM in clinical encounters. Currently, most research explores the impact of the intervention (SDM) on health outcomes, but not as SDM as an outcome itself, which we suspect may vary depending on the healthcare provider, healthcare setting and patient’s conditions. It is important to understand in what settings and for what conditions SDM is most appropriate/effective, which is why we have now decided to explore the impact of anxiety/depression, and HRQL on the level of SDM.

As such, it should be clarified that this study is not only including individuals with A/D. Additionally, it may help clarity of the paper to provide additional information about what conditions the participants are presenting for and to note clearly that, per consort chart, a majority (approx. 76%) of participants are in mental health versus primary care settings. The title may imply even numbers of participants in these settings.

We have clarified in the text of the manuscript that the same is among emerging adults with and without anxiety and depression.

5. It is recommended that the conclusion of the paper be rewritten to more clearly outline the results and their implications. Importantly, the current interpretation of results in the discussion includes a number of statements that may not be supported by the evidence. First, they state the negative relationship between anxiety/depression and PHE implies that promotion of PHE could help reduce symptoms of depression and anxiety. This is a correlational relationship, and it seems just as likely that reducing symptoms of depression and anxiety may increase PHE.

Thank you, we removed PHE from the analysis this variable was not necessary for addressing our research question.

Second, the authors note that their findings are consistent with other studies suggesting a relationship between anxiety/depression and PHE, but also state they are the first study on this topic. It should be clarified how the current study fits into the existing literature. There are also a couple of times throughout the conclusion when related literature is described but it is not clear how this relates to the current study. For example, the authors suggest that cognitive impairment may be one reason for relationship between PHE and anxiety/depression and it is unclear why only one variable was highlighted, since cognitive impairment was not studied or discussed in the background literature. Similarly, the 4th paragraph of the conclusion begins with a topic sentence discussing PHE/SDM but the rest of the paragraph does not refer to SDM.

Thank you, we agree other studies have been conducted around shared decision-making in mental health but this study is novel as it explores predictors of shared decision-making in emerging adults with primary care or mental health settings. This has not been done before to our knowledge.

6. The study flow chart and tables are particularly helpful for understanding the study participants and analysis/results. It would be helpful to relabel table so include more specific labels than the “value” at the top. Additionally, percent signs may be helpful and df may be included for the Chi squared analysis.

Thank you, as the n (%) are located on the columns to the left and not every row reports the same statistic, that “value” is the most appropriate name for the right column on table 1. Since we have now reran the analysis using continuous values we are no longer using the chi-square test.

Some more minor points are as follows:

- In the background of the abstract (page 3), the authors state people with anxiety and depressive disorders may respond better to SDM approaches. It should be clarified whether the authors are saying that they respond better to SDM approaches as compared to other approaches or as compared to other populations.

Thank you, we have tried to clarify this and state that people respond better to SDM compared to conventional decision-making approaches, and it is unknown what population may respond best to SDM.

Revised text:

*“Shared decision-making (SDM) is a process in which healthcare providers (HCPs) and patients make health-related decisions collaboratively, guided by the best available evidence. Previous research suggests emerging adults (18-29 years) with mental health concerns may prefer SDM compared to conventional approaches; however, it is unclear whether prevalent symptoms of anxiety, depression and health-related quality of life (HRQL) are associated with the level of SDM that occurs during a clinical encounter.”*

- The description of 31 healthcare providers and 42 emerging adult patient dyads may be more clearly described as: “42 dyads (31 unique healthcare providers)”

Thank you, we have revised the participant characteristics section to reflect this.

- The keywords for the article do not seem to reflect the major point of the paper. For example, it is recommended that shared decision-making and patient engagement (versus patient centered care) are included as keywords. Anxiety and depression should be included only if this is key to the authors research questions (see comments above).

Thank you for this. We have made the suggested edits.

*Revised text: “shared decision-making primary healthcare, mental health, anxiety, depression, depressive disorder, young adult”*

- On page 4, the authors cite a recent systematic review that suggests that SDM may benefit adults with anxiety and depression. Please clarify in what way SDM benefits.

Thank you, we have now further elaborated on this study.

Revised text:

*“Shared decision-making (SDM) is a clinical decision-making process in which patients and healthcare providers (HCPs) make collaborative and evidence-informed health-related decisions such as goal setting and selection of treatment options [1]. Experts consider SDM to be an essential aspect of person-centred care, which prioritizes patients’ preferences and needs [2]. Evidence from systematic reviews suggests SDM may improve the likelihood of obtaining evidence-based treatment and may enhance patient-reported outcomes such as therapeutic alliance (i.e., trust), treatment engagement or adherence, including in adults with mental health concerns such as anxiety and depression [3-6].”*

-

On page 4, the authors refer to “younger individuals” with mental illnesses. Please pick one term, such as “emerging adults” to use throughout the introduction for clarity.

Thank you, we have omitted this text as we have reworked our research questions and have revised the background considerably. We have also checked the rest of the manuscript to ensure we are consistent in using “emerging adult” instead of “young adult”, “young people”, etc.

- The limitations section should only include limitations of the study and the first paragraph indicating “strengths” of the study can be cut

Thank you, we have omitted the strengths paragraph and renamed the section to “limitations”.

**Reviewer #2:**

Shared decision-making is gaining prominence in the mental health field, and I found the manuscript to be quite captivating. Below, I have outlined my comments regarding the key points I observed. I hope that this will be valuable for enhancing the paper.

1. The introduction made it clear that the primary focus was on examining the relationship between Shared Decision-Making (SDM) and factors such as anxiety or depressive symptoms, patient health engagement, and health-related quality of life. However, the hypotheses formulated by the authors in this study were not clearly stated. Please articulate the hypotheses in this study clearly and provide a coherent explanation for the necessity of these hypotheses."

Thank you, in light of these and the other reviewer’s comments, we have decided to clarify the research questions and more clearly report our hypotheses as follows:

*“The primary objective of this study was to explore whether prevalent symptoms of anxiety, depression or HRQL were associated with the level of dyadic SDM reported after a clinical encounter among emerging adults who obtained health services in primary care or addiction and mental health (AMH) settings in Alberta, Canada.* *We hypothesized that symptoms of anxiety or depression would be negatively associated with SDM (i.e., increased symptoms would yield decreased SDM ratings), and HRQL would be positively associated with SDM (i.e., increased HRQL would yield increased SDM ratings). In a post-hoc analysis, we explored whether patient age was associated with dyadic SDM. Here, we hypothesized age would be negatively associated with SDM.”*

2. It was mentioned that 31 healthcare professionals and 42 patients participated. How many individuals declined to participate? How many individuals did not meet the inclusion criteria and were excluded? I propose providing a detailed description of how many individuals were approached for both healthcare professionals and patients, and as a result, 31 healthcare professionals and 42 patients were included.

Thank you, we agree this would be helpful to know, but unfortunately, we were not able to collect this information in detail. Healthcare providers were asked first, who then asked their patients if they would like to participate. Some healthcare providers at busy centres declined to participate, so we cannot rule out any sampling bias as providers who were more interested in SDM may have been more willing to participate in the study. There also may have been a risk of performance bias, since clinicians knew they were going to be evaluated on SDM and, therefore ensured they conducted SDM to their best ability in the study clinic visit.

Revised text:

*“Several limitations in this study warrant a cautious interpretation of the results and limit the generalizability of the findings. First, a cross-sectional design cannot ascertain a temporal or causative relationship between the study variables [34]. Secondly, a small sample size was obtained as relatively few individuals aged 18-29 participated in the larger study, consistent with the generally low rates of healthcare utilization among emerging adults [9]. The small sample size precluded comparing outcomes between primary care and community addiction and mental health settings, which may be of interest in future research. Incomplete demographic data precluded exploring additional variables such as gender identity, race/ethnicity, and socioeconomic status. Additionally, participants (patients and HCPs) who were more likely to value an SDM approach may have been more willing to participate in the study, leading to selection and performance biases, particularly as the number of individuals who declined to participate could not be reliably collected. A ceiling effect was observed on the SDM dyad ratings, which may be due to a performance bias from the study participants, who may have conducted a clinic visit strictly according to the SDM criteria in the research study (Hawthorne effect), which may not occur in a real-world setting. The lack of longitudinal data precluded any ability to observe the possible associations of study variables on SDM over time. Larger longitudinal studies may help assess the barriers and facilitators of SDM in patients with mental health concerns.”*

3. I understood that pages 8, lines 1 to 6, contain descriptions regarding the responses of dyads, referring to pairs of patients and healthcare professionals. However, in Table 3, apart from these dyad results, the top two rows display results for 31 healthcare professionals and 42 patients. I am curious about the relationship between the top two rows in Table 3 (results for 31 healthcare professionals and 42 patients) and the third row (results for Dyads of patients and healthcare professionals). It would be helpful for better understanding if you could explain the details of what is presented in Table 3 in the text, row by row.

Thank you, as we have decided to measure SDM as a continuous variable as suggested by reviewer one. The results of Table 3 have now changed and include a correlation matrix. We have now reported the results of this table line by line as suggested below.

4. On page 8, in the 9th line, it is stated 'Table 4 shows the association with PHE and HRQL among emerging adults.' However, upon reviewing Table 4, it is evident that Table 4 reports the association between SDM and anxiety and/or depression, HRQL, and treatment setting, rather than the association between SDM, PHE, and HRQL. Please correct this discrepancy to ensure alignment between the information presented in the Table 4 and the explanation provided in the text."

Thank you, we have omitted this text and revised the results table to include a correlation matrix of all study variables explored in the analyses.

5. Related to this, on page 8, in the 9th line, it states, 'No significant relationship was observed between SDM and PHE, r=0.03, P=0.83.' However, this information cannot be inferred from Table 4.

Thank you, we have omitted this text as we are no longer including PHE in the analysis.

6. There were criteria provided in the main text for categorizing HRQL into 'Normal' and 'Abnormal.' However, for anxiety and depression, there is no explanation in the main text regarding the criteria for categorizing into 'Normal' and 'Abnormal,' yet these classifications are used in Table 4. Please provide an explanation for the criteria in the main text."

Thank you, since we have revised the analysis to use continuous variables per another reviewer's suggestion, we are no longer using the “normal” and “abnormal” classifiers. We have now used the visual analogue scale portion of the HRQL to obtain this measurement.

7. On page 8, in the 15th line, it is stated 'Table 5 shows the association between PHE and HRQL.' However, upon reviewing Table 5, it is evident that the table not only displays the association between PHE and HRQL but also the association between PHE, SDM, and anxiety and/or depression. I propose correcting the description to accurately reflect the contents of the table.

Thank you, we have revised the final results table (now Table 3) to reflect our revised analysis. The new table consists of a correlation matrix of all study variables.

8. In the results, please clearly describe whether the formulated hypotheses were supported or rejected based on the study results.

Thank you, we have rewritten the results section as follows to address the hypotheses more directly:

***“Main findings***

*After performing Pearson R correlations, symptoms of anxiety, depression and HRQL were not significantly correlated with SDM dyad ratings during the clinic visit. However, post hoc analyses revealed that patient age was negatively correlated with SDM dyad ratings; R=-0.34, p=0.03. A sensitivity analysis showed symptoms of anxiety were negatively correlated with HRQL, R=0.50, p<0.001, and symptoms of depression were negatively correlated with HRQL, R=0.61, p<0.001. Table 3 shows the summary of the main findings.”*

Based on that, in the Discussion section, please discuss the factors that contributed to the support or rejection of the hypotheses.

Thank you. Based on the revised research questions/hypotheses and analysis, we have made a major revision to the discussion section. We have now discussed possible factors that may have contributed to the support and rejection of our hypotheses.

Revised text:

*Discussion*

*“To our knowledge, this is the first study to explore whether patient characteristics such as HRQL, and symptoms of anxiety or depression are associated with the dyadic (i.e., patient and HCP reported) level of SDM in emerging adults who obtained care in a community mental health or primary care setting. Contrary to our hypotheses, we did not observe a statistically significant association between symptoms of anxiety, depression or HRQL with SDM. Considering the mean level of dyadic SDM was in the “excellent” range, these results suggest emerging adults in primary care or community addiction and mental health settings, with or without anxiety or depression, may be able to participate in a high level of SDM regardless of their mental health status provided it is not acutely severe (e.g., warranting urgent hospitalization). This study provides preliminary evidence that the level of SDM may not be associated with symptoms of anxiety, depression or low HRQL in community outpatient settings in emerging adults. Our main findings are consistent with previous research suggesting individuals (inclusive of emerging adults) with serious mental illnesses can participate in mental health-related treatment decisions, consistent with an SDM approach [29-31]. Previous research also suggests that adults with depression in primary care may also be able to engage in SDM; however, trust (i.e., therapeutic alliance) between the patients and HCPs may be an essential factor for facilitating SDM [32].*

*To better understand these findings, we conducted post hoc analyses exploring whether additional patient characteristics, such as age, may be associated with the dyadic level of SDM. We found that patient age showed a weak but statistically significant negative (i.e., inverse) association with SDM. The interpretation of the association of age on SDM is unclear, considering only emerging adults were included in the analysis (18-29 years). This finding has not been observed to our knowledge and more research is needed to clarify the role of patient age on preference and ability to engage in SDM.*

6. PLOS authors have the option to publish the peer review history of their article (what does this mean?). If published, this will include your full peer review and any attached files.

If you choose “no”, your identity will remain anonymous but your review may still be made public.

Do you want your identity to be public for this peer review? For information about this choice,

including consent withdrawal, please see our Privacy Policy.

Reviewer #1: No

Reviewer #2: No

[NOTE: If reviewer comments were submitted as an attachment file, they will be attached to this email and accessible via the submission site. Please log into your account, locate the manuscript record, and check for the action link "View Attachments". If this link does not appear, there are no attachment files.]

While revising your submission, please upload your figure files to the Preflight Analysis and Conversion

Engine (PACE) digital diagnostic tool, https://pacev2.apexcovantage.com/. PACE helps ensure that figures meet PLOS requirements. To use PACE, you must first register as a user. Registration is free.

Then, login and navigate to the UPLOAD tab, where you will find detailed instructions on how to use the tool. If you encounter any issues or have any questions when using PACE, please email PLOS at figures@plos.org. Please note that Supporting Information files do not need this step.
