# Supplementary material for: Predictors of shared decision-making among treatment-seeking emerging adults in primary care and community addiction and mental health settings: A cross-sectional study
Source: PLoS One. 2025 Nov 13;20(11):e0336598. doi: 10.1371/journal.pone.0336598 (PMC12614592; doi:10.1371/journal.pone.0336598)
Supplement: Attachment — Submitted filename: Marshall SDM appeal letter PloS One 27 Jan 2024 tm.docx [file pone.0336598.s006.docx]

09-Dec-2024

Dr. Tyler Marshall

8818 91^ST^ NW Edmonton, AB, T6C 3N4

Canada

[Tyler.marshall@ualberta.ca](mailto:Tyler.marshall@ualberta.ca)

Editor-in-Chief

Dr. Emily Chenette

PloS One

1875 Mission Street, Suite 103 #188
San Francisco, CA 94103
United States

Re: Appeal of decision on manuscript PONE-D-23-23994R1 Predictors of shared decision-making among treatment-seeking emerging adults in primary care and community addiction and mental health settings: a cross-sectional study

Dear editors at PloS One,

I hope this letter finds you well. First, I appreciate the time and effort invested by the editorial team and reviewers in evaluating our manuscript “Predictors of shared decision-making among treatment-seeking emerging adults in primary care and community addiction and mental health settings: a cross-sectional study” (PONE-D23-23994R1).

On behalf of the authorship team, I am writing to appeal the decision to reject our manuscript based on the following considerations:

1. Extended timeline: The review process has spanned over 15 months (submitted on

August 10, 2023).

2. Reviewer feedback: Our initial feedback from the reviewers and initial editor was favourable, and we invested substantial time addressing all comments. However, our manuscript was assigned a new editor, and the comments suggest our responses were not fully reviewed in the current decision.
3. Concerns for future delays: Given our investment, restarting with a different journal

would impose further undue delay.

In this appeal, we wish to address the editor’s concerns and respectfully request reconsideration of the decision.

1. Significance and novelty
   Our study is the first to explore the relationship between shared decision-making (SDM), patient health engagement (PHE), and health-related quality of life (HRQL) among emerging adults (18–25 years) in outpatient primary care and mental health settings. While previous research has demonstrated the effectiveness of SDM in mental health contexts, our study contributes novel findings by focusing on an underexplored population, emerging adults, and by investigating associations with validated, clinically relevant measures such as HADS and EQ-5D-5L. These findings address a critical gap in understanding how symptoms of anxiety and depression may relate to PHE and HRQL in this demographic.
2. Conceptualization and evidence from theory
   We used established frameworks for SDM and HRQL to guide our study conceptually, as described in the introduction. Specifically, we highlighted the theoretical linkage between SDM and patient engagement as a pathway to improved health outcomes. We acknowledge that further elaboration on the theoretical foundations may strengthen the manuscript, and we have revised the introduction to more clearly explain this in more detail.
3. Cross-sectional design and methodological choices
   We carefully selected a cross-sectional design to assess relationships between SDM, PHE, and HRQL at a single point in time during clinic visits. This design was appropriate for our objectives and was feasible for engaging with emerging adults in mental health settings who may be hesitant to participate in research. While qualitative methods could provide deeper insights, our primary aim was to quantify relationships and establish preliminary associations. We have now clarified this rationale further in the revised manuscript in the methods section.
4. Research gap and literature review
   The assertion that “not a single study has been conducted on predictors of SDM among emerging adults” stems from our published systematic review of SDM. While SDM has been studied in other populations, our manuscript explicitly highlights the scarcity of research addressing SDM predictors in emerging adults within primary care and mental health contexts. We recognize the need for a stronger framing of this gap and will enhance the introduction with additional references to underscore this point.
5. Psychometric properties of the ASK-MI instrument
   The ASK-MI instrument’s high Cronbach’s alpha values reflect exceptional internal consistency but may suggest redundancy on some of the questionnaire items. We agree that this warrants further discussion and interpretation in the manuscript. We have also now cited additional publications that have used this instrument.

Manhas KP†, Olson K, Churchill K, Vohra S, Wasylak T. Implementation of a novel rehabilitation model of care across Alberta, Canada: A Focused Ethnography. BMJ Open Quality 2021 Mar 23;10(1):e001261. Doi:10.1136/bmjoq-2020-001261

Manhas KP†, Olson K, Churchill K, Miller J, Teare S, Vohra S, Wasylak T. Exploring patient-centredness, communication and shared decision-making under a new model of care: community rehabilitation in Canada. Health & Social Care in the Community 2021;30(3):1051-1063. https://doi.org/10.1111/hsc.13304

Manhas KP†, Olson K, Churchill K, Faris P, Vohra S, Wasylak T. Measuring Shared Decision-Making and Collaborative Goal Setting in Community Rehabilitation: A Focused Ethnography Using Cross-Sectional Surveys in Canada. BMJ Open 2020;10(8):e034745. doi:10.1136/bmjopen-2019-034745

Manhas KP†, Olson K, Churchill K, Vohra S, Wasylak T. Experiences of shared decision-making in community rehabilitation: a focused ethnography. BMC Health Services Research. 2020 April 19; 20(1):329: https://doi.org/10.1186/s12913-020-05223-4

6. Sample size and generalizability
   While our sample size is modest, it aligns with typical feasibility constraints in clinical research involving emerging adults and outpatient settings. The observed lack of variability in SDM ratings is noted as a limitation in the discussion, and we remain transparent about the study's generalizability.
7. Interpretation of results
   We acknowledge the importance of robust statistical interpretation and are prepared to refine and expand the discussion of our results to address this feedback. Specifically, we can provide greater depth in explaining the lack of variability in SDM ratings and the significance of the associations between anxiety, depression, and HRQL.

We sincerely appreciate the opportunity to contribute to PloS One and believe our manuscript offers meaningful insights that will resonate with the journal's readership. We kindly request a reconsideration of the decision.

Thank you for considering this appeal,

Sincerely,

Tyler

Tyler Marshall PhD MPH

Lead author on behalf of all authors

University of Alberta
