# Supplementary material for: Predictors of shared decision-making among treatment-seeking emerging adults in primary care and community addiction and mental health settings: A cross-sectional study
Source: PLoS One. 2025 Nov 13;20(11):e0336598. doi: 10.1371/journal.pone.0336598 (PMC12614592; doi:10.1371/journal.pone.0336598)
Supplement: Attachment — Submitted filename: Plos One Response to Reviewers PSDM Marshall Oct 3 2025.docx [file pone.0336598.s007.docx]

We thank the editors and reviewers for their careful review of our manuscript and for the constructive feedback. We appreciate the opportunity to revise further and clarify our work. Below, we provide our responses to each comment point-by-point.

**Editor’s Comments**
“We note that there is identifying data in the Supporting Information file < SDM Emerging Adult Minimum Dataset July 23 2024.xlsx >. Due to the inclusion of these potentially identifying data, we have removed this file from your file inventory… Please remove or anonymize all personal information, ensure that the data shared are in accordance with participant consent, and re-upload a fully anonymized data set.”

**Authors’ Reply:**
We thank the editor for this guidance. The dataset was approved for sharing by the University of Alberta's Research Ethics Board. To further reduce the risk of participant identification, we have grouped participant ages into ranges (e.g., 20–24, 25–29, etc.) and confirmed that all other variables (e.g., HADS scores, EQ5D, ASK-MI scores) are non-identifying. Patient IDs are sequential study IDs with no connection to hospital or system records. This de-identified dataset is now safe for public sharing in accordance with participant consent and our ethics protocol.

“Please include a copy of Table 1–3 which you refer to in your text on your paper.”

**Authors’ Reply:**
We will add Tables 1–3 to the revised manuscript in addition to uploading them separately to ensure that all text references correspond directly to the tables provided.

**Reviewer #4**
“The study uses a dyad model in a situation where both the examiner and patient are aware of the results being studied. As mentioned in your limitations, it might introduce an amount of bias in the sample that it is difficult to read the significance of the results.”

**Authors’ Reply:**
Thank you for this comment. To clarify, both participants and clinicians completed separate questionnaires and were not aware of each other’s responses. This design reduces the potential for direct response bias. However, we recognize that the dyadic model itself may still introduce subtle forms of bias. In the revised manuscript, we have strengthened the limitations section to acknowledge this and clarify how it may affect the interpretation of the findings.

“I think it would help to give more information about the ASK-MI questionnaire and what it measures including an example of the same to help clarify what it being measured.”

**Authors’ Reply:**
We thank the reviewer for this suggestion. In the revised manuscript, we have provided a more detailed description of the ASK-MI, including its purpose, structure, and administration. The ASK-MI includes parallel questionnaires for both participants and clinicians, which are completed independently. This design allows us to compare perceptions of shared decision-making without either party being aware of the other’s responses. We will also attach a supplementary file of each version of the document.

We have added the following text into the manuscript methods section (variables, shared decision-making) to clarify this issue.

“Neither the HCP nor the patient were aware of the results of the corresponding questionnaire.”

“In the introduction, you mention the study being related to emerging adults with Anxiety/ Depression and their need/preference for SDM and/or their ability to participate in SDM. In the results however, if I'm understanding correctly, the ASK-MI questionnaire assesses their perception of SDM rather than their preference or necessarily ability but rather the HCPs involvement in engaging in SDM with each patient- which could also be the reason why there was no significant difference between the groups.”

**Authors’ Reply:**
We appreciate this observation. We have clarified throughout the introduction and discussion that our study measured participants’ perceptions of HCP engagement in SDM, rather than their preference for or ability to engage in SDM. We have highlighted this distinction in our interpretation of the findings.

**Reviewer #4**
“It makes sense that patients with current Anxiety/ Depression with a certain HADS cut off score would perceive that their quality of life is worse, but would they perceive that the SDM is worse even if the HCPs provided the same amount of effort to it seems like the question being asked.”

**Authors’ Reply:**
Thank you for this thoughtful comment. Our initial hypothesis was that patients with higher anxiety and depression would be less likely to engage in SDM, even when providers made efforts to support it. Our findings, however, suggest that involvement in SDM may be more complex than patient symptom burden alone. It is possible that healthcare provider engagement plays a critical role in shaping patients’ perceptions of SDM, and that factors such as the amount of time spent with patients or whether they were first-time versus returning patients may also influence these perceptions. We have expanded the discussion to highlight these possibilities and to note that SDM may not be solely determined by patient characteristics, but also by contextual and relational factors.

**Reviewer #4**
“Overall, with these limitations, the validity of the findings in a curtailed sample size remains questionable. I think it would at the least, need a clarification of the above mentioned things.”

**Authors’ Reply:**
We agree that sample size is a limitation and will expand our discussion to explicitly acknowledge its impact on the generalizability and validity of the findings. However, the larger issues may be more related to low natural variability in SDM scores (mostly high ratings). The clinical settings we picked tended to have longer appointments with patients (often 30 mins to 1 hour) which may enable the perception of more SDM occurring. Preexisting awareness of the concept being studied also likely encouraged participants (clinicians) to “perform well”, leading to high scores. More work is needed in future research to limit these sources of bias to observe more natural variability on SDM being done.

**Reviewer #3:**
“All comments have been addressed.”

**Authors’ Reply:**
Thank you
